# Clinical Reports

**Published:** 1889-02

**Authors:** 


### CLINICAL REPORTS.

### SURGICAL CLINIC, COLLEGE OF PHYSICIANS AND SURGEONS, CHICAGO.

Reported by H. S. Warren, M.D.

Case I.—Carcinoma of the cervical region; operation; two secondary hæmorrhages; recovery.

Lawson Clay, aged 23; single; family history, good. Previous history, never sick. Present condition.—About one year ago a tumor was noticed, beginning a little below and behind the right ear, at the posterior border of the sterno-mastoid muscle. At first it seemed to be as large as a hen's egg, but during the last six months it has grown more rapidly than before. The present extent of the tumor is from the mastoid process downward about three inches; laterally about two inches. At first it was quite movable, but now is only slightly movable laterally, it being impossible to move it vertically. It is about as hard as cartilage and apparently consists of one large and two smaller tumors. There is nothing to be felt inside the mouth. Patient has lost about 25 pounds in weight during the last year.

*Operation* by Professor Fenger, assisted by Dr. Davis, Dr. Pearce and Dr. Warren, at the College of Physicians and Surgeons, Nov. 9, 1888. First an incision was made posterior to the sterno-mastoid muscle, extending from the mastoid process to the sterno-clavicular articulation. The tumor was dissected out from below upwards, and was found intimately connected with the sterno-mastoid muscle, and in one place attached to the common carotid artery. In dissecting the tumor out, commencing from below and working upward, first the internal jugular vein was found running directly through the tumor. This, with the facial vein, was ligated at the lower border of the tumor; then, in dissecting up, the common carotid was found attached to the tumor. The attachments of the carotidwere severed without injury to the artery.

Going still farther with the removal of the tumor, it was found to extend into the jugular fossa, necessitating the division of the internal jugular again, this time in the fossa, but it was found impossible to ligate it owing to the extent of growth of the carcinomatous mass in the fossa; an artery forceps was attached to the vein to be left till a provisional closure of the vessel had occurred. The jugular was divided and the tumor removed.

The wound was now dressed antiseptically. First iodoform gauze was packed around the artery forceps, the wound sutured and an antiseptic dressing applied. After 48 hours there was slight elevation of temperature, but the patient was doing well. Seventy-two hours after the operation the wound was dressed by Dr. Warren. There was a slight serous discharge on the dressings, but the wound looked very well. There seemed to be no sepsis.

Four days after the operation *secondary hæmorrhage occurred.* The dressings were first noticed to be slightly colored with blood. The patient was watched closely, and it was noticed that there was oozing of blood from some point. Professor Fenger was called and opened the wound, exposing the carotid artery. Blood was slowly coming through the walls of the external carotid artery near the bifurcation with the internal carotid, necessitating ligation and division of the common carotid, and of the external carotid above its perforation.

The artery forceps left in the jugular fossa at the time of the operation, were now removed, and the wound sutured and dressed.

The patient did very well until November 23, ten days after the second operation, and the removal of the artery forceps, when a *second secondary hæmorrhage* occurred. On this day the patient became suddenly very weak, and called for help. Dr. Warren was near and noticed profuse hæmorrhage coming through the dressings. He at once made firm pressure on the dressing above and below, and the hæmorrhage ceased. Then the dressing was removed. The hæmorrhage this time came from the jugular vein in the jugular fossa, due to separation of the blood clot that was formed by the aid of the artery forceps. A fine compress of iodoform gauze was put over and near the fossa, bound tightly with a roller bandage. The wound was now dressed, and the patient closely watched, but he continued to improve after this, until now, January 14, 1889, he is perfectly well.

The loss of blood at each secondary hæmorrhage was about four ounces. There were at no time any bad symptoms from the ligation of the blood-vessels on that side of the neck.

Case II.—Excision of the Head of the Humerus for Old Dislocation.

Geo. McMurry, aged 56.

*Diagnosis.* — Old dislocation of the humerus at the shoulder joint. Family history good. Present condition.—About nine months before coming to the College, the patient fell from a wagon, striking the tongue of it with his right shoulder, causing a downward and forward dislocation of the shoulder-joint. A few minutes after the injury paralysis of the motor nerves of arm occurred with sensory paralysis on the extensor surface of arm. No attempt was made at reduction until he came to the College, as there was no physician within 40 miles of his home.

*Examination.*—The shoulder showed a disappearance of the humeral prominence, and a marked depression anterior and exterior to the coracoid process. There was inability to rotate the arm, loss of motion, and semi-flexion of the digits due to paresis.

*Operation.*—By Prof. Fenger and assistants. The patient was anæsthetized and an attempt made at reduction, but this was found impossible, owing to adhesions. An incision was now made through integument covering the coracoid process and glonoid cavity. Hæmorrhage was controlled, and the muscles were operated until the osseous structures were reached. An attempt was made to limit the operation to the outside of the long head of the biceps tendon, but was unsuccessful owing to anatomical difficulties. On opening the capsule the glonoid cavity was found filled with newly formed connective tissue which was removed. The coracoid process was then broken to give space for the reduction. The head of the humerus was found diseased, making removal necessary a little below the anatomical neck. The end of the humerus was then inserted into glonoid cavity and a drainage tube inserted. The wound was now closed with silk sutures. An antiseptic dressing was put on with a plaster cast covering the shoulder and chest. The patient at no time after the operation developed much temperature. There was no sepsis, and the patient improved rapidly. The result was good, there being primary union of the wound.

The paralysis disappeared. The function of the joint was restored, and the patient went home with a fairly useful arm.

---

### CLINICAL NOTES FROM COOK COUNTY HOSPITAL.

I. Secondary Epithelioma of Cervical Lymph Glands: Resection of Lower Jaw.

John K—, white, aged 49 years, married, was admitted on November 16th. His past history reveals nothing that has any bearing on present complaint. His father died from sunstroke, his mother from old age.

His present trouble dates back three months, when he first noticed a small lump on the right side of the neck, just anterior and internal to the angle of the jaw. It was about as large as a hazel nut, movable, painless, and of moderately firm consistence. From that time it has gradually increased in size, but has never pained him until two weeks ago. Since then the pain has been constant, with exacerbations lancinating in character. There has been no constitutional disturbance since the advent of the growth. Appetite is good; throat has not been sore; no interference with respiration or deglutition, and patient has worked up to date.

Upon examining the tumor it was found to be about the size of a goose egg, situated over the body of the inferior maxillary and submaxillary region of the right side, quite firmly adherent to the bone. The most dependent part was semi-fluctuating, the rest rather firm. The skin over tumor was red, and seemed to be closely attached to it. On the left side of the neck there were three or four enlarged glands. The supraclavicular and deep cervical glands were apparently not enlarged. There was also on the lower lip a small ulcer, similar to a "cold-sore," which the patient claimed had only been there a week or two, but which was evidently the primary epithelioma.

*Operation* (Nov. 22). An elliptical incision was made, and the tumor was rapidly loosened from its attachments to the neck by a scalpel-handle. The hæmorrhage was profuse, requiring several ligatures. The connection to the lower jaw was found to be so intimate that a resection was decided upon. Accordingly a central incisor was extracted, and a chain saw passed in the median line *beneath* the mucous membrane (thus preventing any blood being swallowed), the bone sawn through, and again just in front of the coronoid process. After a few cuts with scissors the tumor and resected bone were detached. A blind rubber tube was inserted, and the wound closed by deep and superficial sutures.

Nov. 23. Temperature 99° F. There had been considerable oozing, so the dressing was changed.

Nov. 29. Deep sutures loosened.

Nov. 30. All sutures removed. Owing to large loss of tissue the wound had to heal by granulation. But a return of the disease occurred at the site of operation, and the patient left on December 31st, unimproved.

II. Chronic Synovitis; Amputation; Death From Multiple Secondary Hæmorrhages.

Thomas K——, white; aged 44 years; single; was admitted December 10th, 1887. His family history was obscure, but his previous health had been good, with the exception of trichiniasis some nineteen years previous in Germany.

His occupation for many years was that of a laborer in a packing-house, where the floors were constantly wet and the air warm and moist. In 1885 he noticed that it pained him to flex his knees. In a month the pain had increased so that he was obliged to quit work, and the affection grew steadily worse. On admission he could walk a block at a time, but had to go very slowly. In going upstairs he had to step up with the left leg and draw the right one after. Though both knees were affected simultaneously, the right knee had always occasioned the most discomfort. Has decreased in weight 60 pounds since advent of disease. His sleep was disturbed by sudden jerks of the muscles of the leg, causing great pain. Any movement of the knees pained him, and rest in any one position was unbearable for more than half an hour.

On examination the right leg was found flexed on the thigh, and the knee-joint symmetrically swollen. The part over the femur was puffy, and did not pit or fluctuate. Lower down was harder, and small loose bodies could be felt, slipping under the finger. The leg could be extended to nearly the full distance, and there was no creaking on moving it. No pulsation or bruit could be detected. The left knee was similarly affected, but the swelling was smaller, and a grating sensation was plainly felt on moving the leg.

Various local applications were used with no appreciable effect, and on December 16, extension with one brick was tried, and kept up till the 28th, with a similar result.

On December 28, excision of the left knee was decided on. The usual incision was made, and the joint-cavity exposed, when the synoival membrane, except that covering the trochlear surface of the femur, was found studded with small polypi or granulations. Clots of blood in various stages of disorganization extended some four inches up the thigh under the quadriceps. The cartilages were eroded. Owing to the latter features an amputation seemed to promise better results; a circular amputation was made in the lower third of the thigh. On cutting through the muscles they were found thickly studded with small yellow bodies, which on microscopical examination proved to be encapsulated trichinæ completely calcified. The arteries were all atheromatous, and considerable difficulty was experienced in ligating the larger vessels. The femoral artery and vein were tied in two places with juniper catgut.

December 29. Temperature 99.5° F. Hæmorrhage was so profuse that dressing had to be taken off and the clots turned out. The patient was stimulated constantly, but on January 4, a large amount of clotted blood had to be taken out again, the hæmorrhage checked with hot water, the wound packed with iodoform gauze, and dressed open.

January 12. Another hæmorrhage occurred, from which the patient never rallied, and died that night.

